# Using Transcriptomics to Determine the Mechanism for the Resistance to Fusarium Head Blight of a Wheat-*Th. elongatum* Translocation Line

**DOI:** 10.3390/ijms25179452

**Published:** 2024-08-30

**Authors:** Yi Dai, Wenlin Fei, Shiqiang Chen, Juntao Shi, Haigang Ma, Haifeng Li, Jinfeng Li, Yonggang Wang, Yujiao Gao, Jinghuan Zhu, Bingkui Wang, Jianmin Chen, Hongxiang Ma

**Affiliations:** 1 Jiangsu Key Laboratory of Crop Genomics and Molecular Breeding/Key Laboratory of Plant Functional Genomics of the Ministry of Education/Jiangsu Key Laboratory of Crop Genetics and Physiology, Yangzhou University, Yangzhou 225009, China; daiyi@yzu.edu.cn (Y.D.); mz120221750@stu.yzu.edu.cn (W.F.); sjt0602@163.com (J.S.); mahg@yzu.edu.cn (H.M.); mz120211652@stu.yzu.edu.cn (J.L.); yg.wang@yzu.edu.cn (Y.W.); yujiaogao@yzu.edu.cn (Y.G.); jmchen@yzu.edu.cn (J.C.); 2 Joint International Research Laboratory of Agriculture and Agri-Product Safety, The Ministry of Education of China, Yangzhou University, Yangzhou 225009, China; 3 Jiangsu Co-Innovation Center for Modern Production Technology of Grain Crops, Yangzhou University, Yangzhou 225009, China; 4 Institute of Agricultural Sciences for Lixiahe Region in Jiangsu, Yangzhou 225009, China; 5 College of Bioscience and Biotechnology, Yangzhou University, Yangzhou 225009, China; haifengli902@163.com; 6 Institute of Crops and Nuclear Technology Utilization, Zhejiang Academy of Agricultural Sciences, Hangzhou 310021, China; jinghuanz@163.com (J.Z.); bingkuiwang2006@126.com (B.W.)

**Keywords:** Fusarium head blight, wheat-*Th. elongatum* translocation line, transcriptome analysis, disease resistance pathway

## Abstract

Fusarium head blight (FHB), caused by the *Fusarium graminearum* species complex, is a destructive disease in wheat worldwide. The lack of FHB-resistant germplasm is a barrier in wheat breeding for resistance to FHB. *Thinopyrum elongatum* is an important relative that has been successfully used for the genetic improvement of wheat. In this study, a translocation line, YNM158, with the YM158 genetic background carrying a fragment of diploid *Th. elongatum* 7EL chromosome created using ^60^Co-γ radiation, showed high resistance to FHB under both field and greenhouse conditions. Transcriptome analysis confirmed that the horizontal transfer gene, encoding glutathione S-transferase (*GST*), is an important contributor to FHB resistance in the pathogen infection stage, whereas the 7EL chromosome fragment carries other genes regulated by *F. graminearum* during the colonization stage. Introgression of the 7EL fragment affected the expression of wheat genes that were enriched in resistance pathways, including the phosphatidylinositol signaling system, protein processing in the endoplasmic reticulum, plant–pathogen interaction, and the mitogen-activated protein kinase (MAPK) signaling pathway at different stages after *F. graminearium* infection. This study provides a novel germplasm for wheat resistance to FHB and new insights into the molecular mechanisms of wheat resistance to FHB.

## 1. Introduction

Fusarium head blight (FHB) is a primary disease caused by pathogens such as *F. asiaticum* and *F. graminearium*, which seriously affects the yield and quality of wheat worldwide. In addition to causing a substantial reduction in yield, FHB leads to the accumulation of the toxin, deoxynivalenol (DON), in the seeds of infected wheat, which poses a risk to food safety. With global warming and changes in farming systems and practices, there is a tendency to expand the occurrence of wheat FHB; the cultivation of FHB-resistant varieties is a fundamental way to reduce this damage. However, the sources of resistance to wheat FHB are relatively limited. There are only eight quantitative trait loci (QTLs) (*Fhb1* to *Fhb8*) related to FHB resistance, including *Fhb1* from chromosome 3BS and *Fhb2* from chromosome 6BS of Sumai3 [1,2], *Fhb4* from chromosome 4B, *Fhb5* from chromosome 5A, and *Fhb8* from chromosome 7D of Wangshuibai [3,4,5]. *Fhb1* is recognized as the most stable master-effect QTL for FHB-resistance expansion and is widely used in wheat breeding for FHB resistance. Furthermore, QTLs are also found in wheat relatives, such as *Fhb3* from *Leymus racemosus* [6], *Fhb6* from *Elymus tsukushiensi* [7], and *Fhb7* from *Thinopyrum ponticum* [8,9].

As a close relative of wheat, tall wheatgrass has many important favorable traits that represent a valuable source of alien gene resources for wheat. There are three tall wheatgrass species in nature: *Th. elongatum* (diploid, 2n = 2x = 14; E^e^E^e^), *Th. scirpeum* (tetraploid, 2n = 4x = 28; E^e^E^e^E^b^E^b^), and *Th. ponticum* (decaploid, 2n = 10x = 70; E^e^E^e^E^b^E^b^E^x^E^x^StStStSt). There is no scientific conclusion on the evolutionary process of polyploidy in the family of *Thinopyrum*, but it is believed that many interspecific hybridizations and natural doubling of chromosomes occur during the evolutionary process of tall wheatgrass, which is similar to the situation in common wheat [10]. As tall wheatgrass has strong resistance to wheat FHB, breeders have constructed wheatgrass chromosome addition, and substitution lines and other genetic materials by distant hybridization using *Th. elongatum* and *Th. ponticum*, and obtained the results related to FHB resistance. For example, Jauhar et al. created 1E addition lines, 1E(1A) and 1E(1B) diploid substitution lines by crossing durum wheat with diploid *Th. elongatum*, and found that the 1E chromosome of the diploid *Th. elongatum* may carry FHB-resistant genes [11,12]. Liu et al. [13] obtained a disomic alien addition line by using a pair of 7E *Th. scirpeum* chromosomes by hybridization of durum cultivar, “Langdon”, with the amphiploid 8801 (2n = 6x = 42, AABBEE) and found this addition line showed high resistance to FHB. Shen et al. [14] identified strong FHB resistance in the Chinese Spring *Th. elongatum* substitution lines, 7E(7A), 7E(7B), 7E(7D), and the Thatcher-*Th. ponticum* substitution line, 7el_2_(7D).

The FHB resistance locus, *FhbLoP*, was mapped to the distal region of the long arm of chromosome 7E in *Th. ponticum* within a 3.71 cm interval flanked by *Xcfa2240* and *Xswes19*, accounting for 30.46% of the phenotypic variance [15]. This locus was designated as *Fhb7* and fine-mapped at a 1.7 cm interval [8]. Subsequently, the *Fhb7* gene was successfully cloned from Th. ponticum 7el_2_ by assembling the genome of diploid *Th. elongatum* [9]. The authors demonstrated this gene encoded a glutathione S-transferase (GST), which can open the epoxy group of the DON toxin and catalyze the formation of glutathione adduct (DON-GSH), resulting in detoxification and anti-FHB effects. However, Guo et al. [16] discovered that some wheat-*Thinopyrum* derivatives carrying *Fhb7* homologs reacted differently in FHB resistance. Similar results have been observed in transgenic plants overexpressing GST-encoding *Fhb7* [17]. Regardless of the species (*Th. elongatum*, *Th. scirpeum* or *Th. ponticum*), FHB-resistance sites have been reported on homologous group seven of the E genome, which can be used in wheat FHB resistance breeding [13,18]. For example, Zhang et al. [19] incorporated a novel *Fhb7* allele, *Fhb7^The2^*, into the wheat B genome through a small 7B–7E translocation (7BS·7BL–7EL) involving the terminal regions of the long arms, making this novel FHB resistance allele usable for breeding in both common and durum wheat.

It is well known that the introduction of chromosomes from wild species into wheat usually results in linkage drag of undesirable genes, which limits their application. In wheat genetic improvement, breeding translocation lines carrying alien beneficial genes, especially small fragment translocation lines, can reduce the linkage caused by alien chromosomes and provide high genetic stability under a common wheat genetic background. Therefore, in this study, we aim to obtain translocation lines with different sizes of 7EL chromosome fragment, develop a stable inherited translocation line with FHB resistance, evaluate the application of translocation lines in wheat breeding for FHB resistance, explore potential disease resistance genes, and analyze the disease resistance pathways in translocation lines using transcriptome analysis. The results not only provide a new germplasm for wheat FHB resistance breeding but also a theoretical basis for studying the resistance mechanism of wheat FHB.

## 2. Results

### 2.1. Establishment of a Wheat-Th. elongatum 7EL Chromosome Translocation Line YNM158 with FHB Resistance

**Figure 1:**
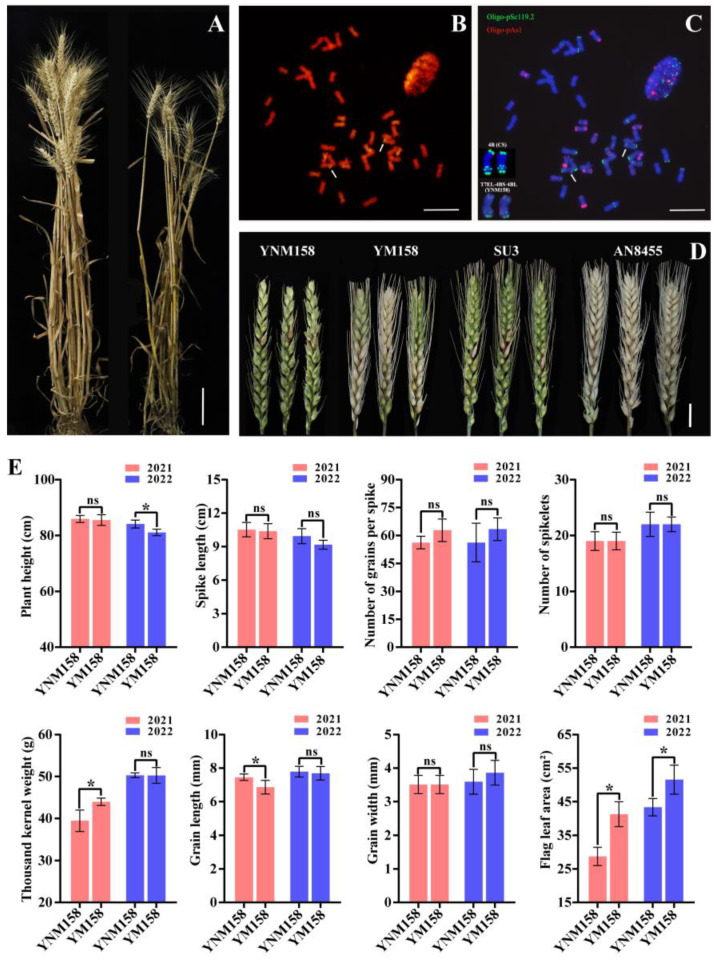
The establishment of wheat-*Th. elongatum* 7EL chromosome translocation line YNM158. (**A**) Mature plants of YNM158 (left) and YM158 (right). Scale bar = 10 cm. (**B**) GISH analysis of the translocation lines YNM158: diploid *Th. elongatum* genomic DNA was used as a probe (green); arrows show the translocated chromosome pair. Scale bar = 100 μm. (**C**) ND-FISH analysis of the translocation lines YNM158: Oligo-pAs1 (red signal) modified with 5′TAMRA and Oligo-pSc119.2 (green signal) modified with 5′FAM were used as probes; chromosomes were counterstained with DAPI (blue), and arrows show the translocated chromosome pairs. Scale bar = 100 μm. (**D**) Symptoms of YNM158 and the control varieties at 21 dpi with *F. graminearum* isolate F0609. Scale bar = 2 cm. (**E**) Statistical analysis of eight agronomic traits for YNM158 and YM158. Statistical significance of differences was evaluated by *t*-test (* *p* < 0.05).

In order to reduce the negative effects of linkage drag by the alien chromosomes, the male gamete irradiation of T7BS·7EL was used to produce chromosomal aberrations. A total of 94 F_1_ plants derived from the cross between common wheat, YM158, and male gamete-irradiated T7BS·7EL were obtained. By genomic in situ hybridization (GISH), 12 F_1_ plants were found to have chromosomal aberrations involving 7EL and were selected for backcrossing with YM158. Finally, a line with stable agronomic characteristics was obtained from the BC_1_F_6_ generation, which was named YNM158 (Figure 1A). The root-tip cells of YNM158 at mitotic metaphase were analyzed using GISH and fluorescence in situ hybridization (FISH). First, the presence of the translocated chromosome pair was confirmed in YNM158 using GISH (Figure 1B). Chromosomal structural variation was observed at the end of the 4BS chromosome according to the standard karyotype of the CS (Figure 1C). Finally, the translocation chromosome was represented as T7EL–4BS·4BL.

The FHB resistance of YNM158 over two consecutive years showed that YNM158 had high resistance to FHB in both field and greenhouse studies and there was no marked difference in the percentage of diseased spikelets between YNM158 and Sumai3 (SU3) (Figure 1D and Table 1). Moreover, almost no differences in the tested agronomic traits, including spike length, number of grains per spike, number of spikelets, and grain width, were observed over two consecutive years (Figure 1E). Plant height, thousand kernel weight, and grain length of YNM158 were substantially different from those of YM158 at one year (Figure 1E). However, the flag leaf area of YNM158 was smaller than that of YM158 over two consecutive years (Figure 1E).

### 2.2. RNA-Sequencing (RNA-Seq) Data Quality, Assembly, and Annotation of YNM158 and YM158

To analyze the genes associated with FHB resistance on chromosome 7EL in YNM158, RNA-seq-based transcriptome profiling was performed on spikes inoculated with F0609. After filtering out the rRNAs and low-quality reads, a total of 193.57 GB of high-quality clean data were obtained from 24 libraries (BioProject ID: PRJNA1011388), with an average of 80.65 GB of clean data per library. The Q20 and Q30 values were >97% and >93%, respectively. In addition, the GC content was 48.86–52.53% in all samples.

After assembly, the clean reads were mapped to wheat and *Th. elongatum* reference genomes (Chinese Spring v2.1 and *Th. elongatum* v1.0). On average, 91.17% of reads were successfully aligned to the reference genome (Table S1). Therefore, these analyses indicated that the quality of the RNA-seq data was high and the sequencing depth was sufficient for further analysis.

To investigate the impact of chromosomal translocations on gene expression, RNA-seq-based transcriptome profiling was performed on the wheat variety, YM158, which is one of the parents of YNM158. In this study, cDNA libraries (24 cDNA libraries) of YM158 were constructed at different times after *F. graminearum* infection. After filtering out the rRNAs and low-quality reads, a total of 188.56 GB of high-quality clean data were obtained from 24 libraries (BioProject ID: PRJNA1011388), with an average of 78.57 GB clean data per library. The Q20 and Q30 values were >97% and >94%, respectively. After assembly, clean reads were mapped to the wheat reference genome (Chinese Spring v2.1). On average, 89.31% of the reads were successfully aligned to the reference genome (Table S1). Therefore, these analyses indicated that the quality of the RNA-seq data was high and the sequencing depth was sufficient for further analysis.

### 2.3. Identification of the Differentially Expressed Genes (DEGs) on Chromosome 7EL Post Inoculation with F. graminearum

**Figure 2:**
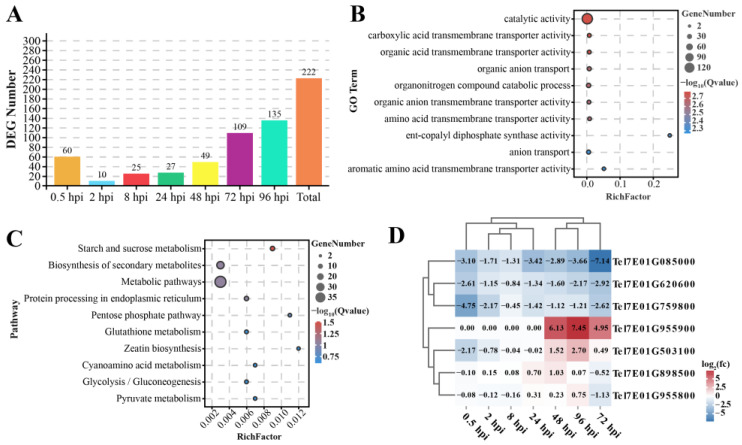
Analysis of the differentially expressed genes (DEGs) identified from YNM158. (**A**) Statistical analysis of the DEGs number on 7EL chromosome at different times after *F. graminearum* infection. (**B**) Gene ontology function enrichment analysis of DEGs on 7EL chromosome after *F. graminearum* infection. (**C**) KEGG pathway enrichment analysis of DEGs on 7EL chromosome after *F. graminearum* infection. (**D**) The heatmap of the DEGs enriched in starch and sucrose metabolism pathway.

Using the criteria of false discovery rate (FDR) < 0.05 and |log2 (fold change)| > 1, a total of 32,102 DEGs that substantially responded to *F. graminearum* infection at different times in YNM158 were detected, of which 222 DEGs were located on the 7EL chromosome (Table S2). A total of 222 DEGs were analyzed. And a total of, 60, 10, 25, 27, 49, 109, and 135 DEGs at 0.5, 2, 8, 24, 48, 72, and 96 hpi, respectively, were identified (Figure 2A). Gene Ontology (GO) function enrichment analysis indicated that these DEGs were enriched in catalytic, carboxylic acid transmembrane transporter, and organic acid transmembrane transporter activity in terms of molecular function. In total, 124 DEGs were enriched in catalytic activity (Figure 2B and Table S3). In terms of biological processes, organic anion transport, organonitrogen compound catabolic processes, and anion transport were the main roles of the enriched genes. Of these, the organonitrogen compound catabolic process had the most enriched DEGs, with a total of 18 (Figure 2B and Table S3). The top 10 GO terms with the lowest Q values were selected to draw a scatter diagram of the enrichment items (Figure 2B).

Kyoto Encyclopedia of Genes and Genomes (KEGG) pathway analysis revealed that the starch and sucrose metabolism pathway is the only one with the Q value less than 0.05 (Figure 2C, Table S4). Moreover, the heatmap of the DEGs enriched in this pathway showed the expression levels of these genes were down-regulated after infection with *F. graminearum*, especially in the first 8 h after infection (Figure 2D). The top 10 pathways with the lowest Q value were selected to draw the scatter diagram of enrichment items in Figure 2C.

### 2.4. Gene Expression Patterns Analysis of DGEs on Chromosome 7EL at Different Infection Time Points

**Figure 3:**
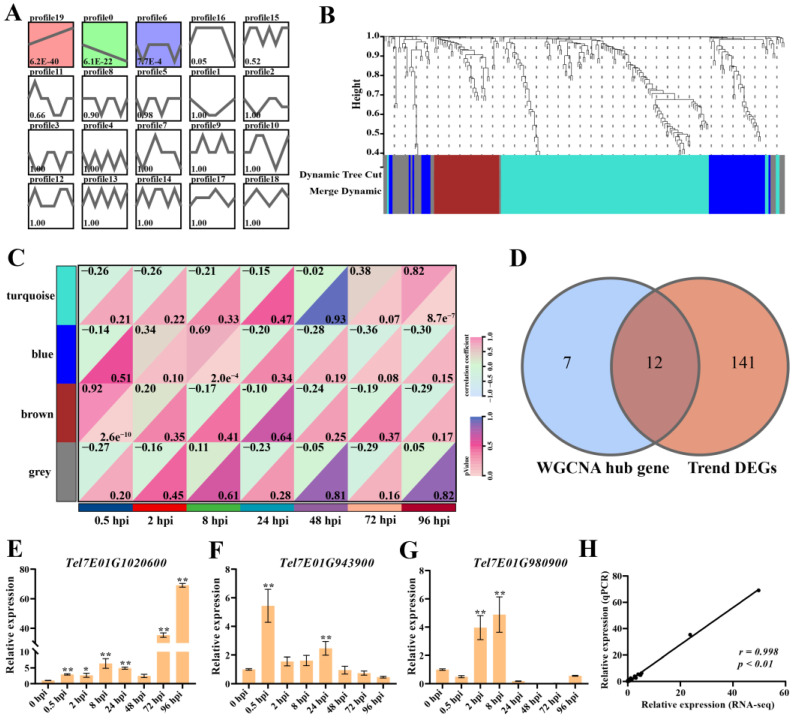
Gene expression patterns analysis on 7EL chromosome. (**A**) The trend analysis of DEGs at different times after *F. graminearum* infection. (**B**) Gene dendrogram by clustering the dissimilarity based on topological overlap. (**C**) Correlation heatmap between modules and infection time with *F. graminearum*. The 4 modules are provided in the left panel. The module–trait correlation, from −1 (light blue) to 1 (pink), is indicated with the color scale on the right. Each column presents the infection time, and their association with each module is represented by a correlation coefficient (showing top-left corner) and a *p*-value (showing lower-right corner). (**D**) Venn diagrams showing the overlapping of DEGs between WGCNA and trend analysis. (**E**–**G**) Relative expression of *Tel7E01G1020600*, *Tel7E01G946300*, and *Tel7E01G980900* by qPCR. (**H**) The correlation analysis between the relative expressions obtained by qPCR and RNA-seq by Pearson correlation analysis. *: *p* < 0.05; **: *p* < 0.01 by Student’s *t*-test.

The analysis of the 222 DEGs on chromosome 7EL showed that the DEGs were clustered into 20 profiles, of which 153 DEGs were clustered to three profiles (profile 19, 0, and 6) on the basis of (*p*-value ˂ 0.05, Figure 3A and Table S5). Of these, 77 genes tended to increase with infection time (profile 19), and 56 genes decreased (profile 0).

In addition, 222 DEGs were analyzed using weighted gene co-expression network analysis (WGCNA) modules associated with infection time. The selection of a soft threshold (power) is a key step in network construction. When the soft threshold was set to 10 with a scale-free network-fitting index of R^2^ > 0.80, the average connectivity was close to zero (Figure S1A). A hierarchical cluster tree was drawn based on the optimal soft threshold, and genes clustered in the same branch were divided into the same module. Finally, four modules (turquoise, blue, brown, and gray) were obtained (Figure S1B). Of them, the brown module was correlated with infection at 0.5 hpi (R = 0.92, *p* ≤ 0.05), the turquoise module was correlated with infection at 8 hpi (R = 0.82, *p* ≤ 0.05), and the blue module was correlated with infection at 96 hpi (R = 0.69, *p* ≤ 0.05). Based on the cut-off criteria, |module membership| (|MM|) > 0.8, and |gene significance| (|GS|) > 0.8, 19 genes with high connectivity in the clinically significant module were identified as hub genes (Figure 3B,C and Table S6).

DEGs and hub genes obtained from trend analysis and WGCNA, respectively, were used to draw Venn diagrams, and 12 genes were identified in both analyses (Figure 3D and Table 2). The quantitative real-time polymerase chain reaction (qPCR) verification of 12 DEGs showed that the relative expression levels of several genes were induced by *F. graminearium*, such as *Tel7E01G1020600*, *Tel7E01G943900*, and *Tel7E01G980900*. Among them, *Tel7E01G1020600* encoded glutathione S-transferase (GST), whose expression was induced by *F. graminearum* and strongly upregulated from 72 hpi (Figure 3E).

*Tel7E01G943900* and *Tel7E01G1980900* showed similar expression patterns and were up-regulated at an early stage of infection. *Tel7E01G943900* encodes a receptor-like kinase, and the expression of this gene was up-regulated by approximately five-fold at 0.5 hpi but then began to be downregulated (Figure 3F). Similarly, *Tel7E01G1980900* encodes a monosaccharide-sensing protein, and the expression of this gene reached its highest level at 2 hpi, which was approximately four times higher than that at 0 hpi but then was downregulated and showed almost no expression after 24 h of infection (Figure 3G). To verify the accuracy of the results, the correlations between the relative expressions obtained using qPCR and RNA-Seq were compared. The expression profiles of these three genes showed similar patterns in qPCR and RNA-seq (*r* = 0.998, *p* < 0.01) (Figure 3H). These results suggest that there may be multiple resistance genes on the chromosome 7EL fragment that provide resistance to FHB at different stages of *F. graminearium* infection.

### 2.5. Extraction of Wheat DEGs between YM158 and YNM158 after F. graminearum Infection

**Figure 4:**
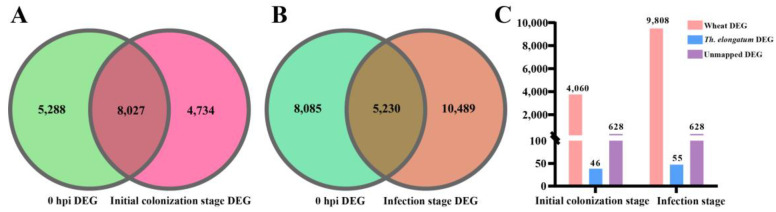
Screened the DEGs between YM158 and YNM158. (**A**) DEGs Venn diagram of initial colonization stage. (**B**) DEGs Venn diagram at infection stage. (**C**) Different types of specific DEGs statistics.

As wheat FHB is a compatible disease, Stephens et al. [20] divided the infection process into initial colonization, infection, and late infection stages. In the current study, the introduction of chromosome 7EL fragments on FHB resistance were analyzed using 8 hpi as the cut-off point. We defined the initial colonization stage as before 8 hpi and the infection stage as after 8 hpi. A total of 12,761 and 15,719 DEGs were identified in the initial colonization and infection stages, respectively (Table S7 and Table S8). The DEGs at 0 hpi between YM158 and YNM158 were removed (Table S9). Finally, 4734 DEGs were obtained at the initial stage of colonization (Figure 4A), and 10,489 DEGs were obtained at the stage of infection (Figure 4B). After alignment with the reference genome, 4060 and 9808 wheat DEGs were screened for subsequent analysis at the initial colonization and infection stages, respectively (Figure 4C and Table S10).

### 2.6. KEGG Pathway Enrichment Analysis of Wheat DEGs at Different Stages

**Figure 5:**
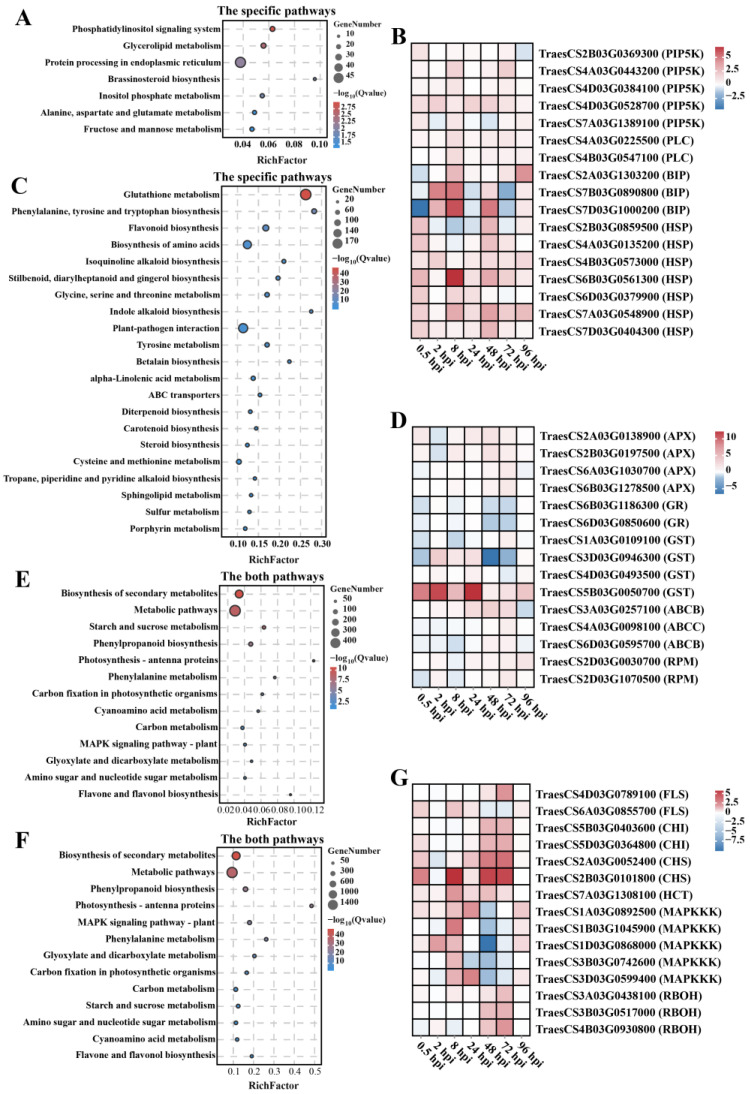
KEGG pathway enrichment analysis of wheat DEGs. (**A**) The specific enrichment pathways at the initial colonization stage. (**B**) The heatmap of the representative DEGs enriched at the initial colonization stage. (**C**) The specific enrichment pathways at the infection stage. (**D**) The heatmap of the representative DEGs enriched at the infection stage. (**E**) The same enrichment pathways at the initial colonization stage. (**F**) The same enrichment pathways at the infection stage. (**G**) The heatmap of the representative DEGs enriched at both stages.

To analyze the effect of chromosome 7EL on the resistance pathway of wheat at different stages of *F. graminearum* infection, the wheat DEGs were subjected to KEGG pathway enrichment analysis. Seven pathways were identified that were substantially enriched (Q value < 0.05) during the initial colonization stage, of which the phosphatidylinositol-signaling system was the most enriched pathway with the lowest Q value; protein processing in the endoplasmic reticulum pathway was the most enriched pathway with the highest number of DEGs (Figure 5A and Table S11). Further analysis of the genes involved in these pathways revealed that the expression of genes related to phosphatidylinositol 4-phosphate 5-kinase (PIP5K), immunoglobulin-binding protein (BIP4), and heat-shock protein (Hsp) in YNM158 was higher than that in YM158 after *F. graminearum* infection (Figure 5B).

At the infection stage, 21 specific pathways were substantially enriched (Q value < 0.05). Of them, glutathione metabolism was the pathway with the lowest Q value and the largest number of DEGs (Figure 5C and Table S11). Moreover, genes related to ascorbate peroxidase (APX), glutathione reductase (GR), glutathione-S-transferase (GST) were up-regulated in YNM158 (Figure 5D). The genes related to the ABC transporter (ATP-binding cassette, ABC) were up-regulated in YNM158 (Figure 5D). The plant–pathogen interaction pathway was the second most enriched pathway, with 152 enriched DEGs (Figure 5C). Using a heat map of gene expression, the expression levels of some genes related to hypersensitive response (HR), such as *TraesCS2D03G0030700* and *TraesCS2D03G1070500*, were found to be upregulated at 24 hpi with *F. graminearum* in YNM158 (Figure 5D).

Secondary metabolites play an important role in plant–pathogen resistance. In this study, 13 pathways were identified in both the initial colonization and infection stages (Q value < 0.05). Of these, the biosynthesis of secondary metabolites was the pathway with the lowest Q value (Figure 5E,F and Table S12). Analysis of gene expression in this pathway revealed that some genes related to flavonol synthase (*FLS*), chalcone isomerase (*CHI*), chalcone synthase (*CHS*), and hydroxycinnamoyl–CoA shikimate (*HCT*) were up-regulated with increasing *F. graminearum* infection time in YNM158 (Figure 5G). In the mitogen-activated protein kinase (MAPK) pathway, the expression of some genes related to respiratory burst oxidase (RBOH) was substantially up-regulated after *F. graminearum* infection, and the expression was the highest at 72 hpi, whereas the expression of some genes related to MAPK were up-regulated at the initial colonization stage and then down-regulated at the infection stage in YNM158 after *F. graminearum* infection (Figure 5G).

### 2.7. WGCNA of Wheat DEGs

**Figure 6:**
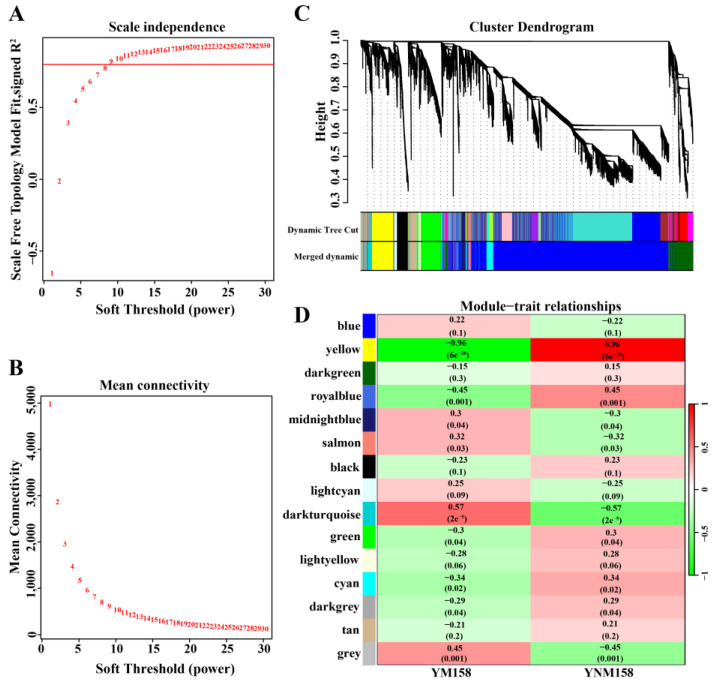
WGCNA of wheat DEGs identified in the YM158 and YNM158 after *F. graminearum* infection. (**A**) The *x*-axis represents the soft threshold β. (**B**) The *y*-axis represents the mean of all genes’ adjacency functions in the corresponding gene module. (**C**) Fourteen modules of co-expressed genes are shown in a hierarchical cluster tree. A major tree branch represents a module. Modules in designated colors are presented in the lower panel. (**D**) Module–trait relationships: The 14 modules are provided in the left panel. The module–trait correlation, from −1 (green) to 1 (red), is indicated with the color scale on the right. The association with each module is represented by a correlation coefficient and a *p*-value (showing in parentheses).

The 12,661 wheat DEGs were analyzed in the WGCNA modules (Table S13). The selection of the soft threshold (power) is a key step in network construction. When the soft threshold was set to nine with a scale-free network-fitting index of R^2^ > 0.80, the average connectivity was close to zero (Figure 6A,B). A hierarchical cluster tree was drawn based on the optimal soft threshold, and genes clustered in the same branch were divided into the same module (Figure 6C). Finally, 14 modules that were correlated with the varieties were obtained. Of these, the yellow module was positively associated with YNM158 (R = 0.96, *p* < 0.05), and 847 DEGs were identified in this module (Figure 6D and Table S14).

### 2.8. Core-Gene Screening at Different Infection Stages

**Figure 7:**
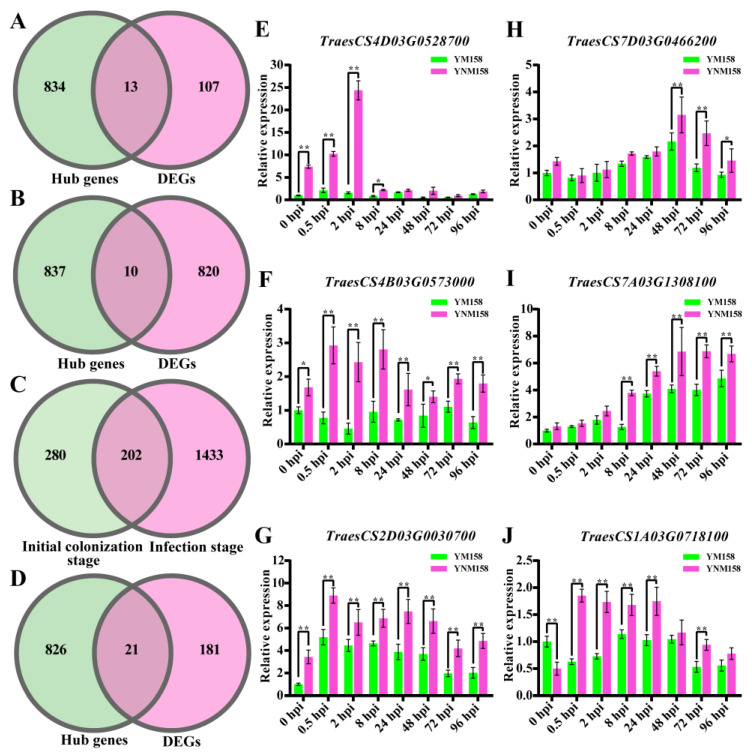
The core genes extraction and expression verification by RT-PCR. (**A**) Venn diagram between the hub genes associated with YNM158 and DEGs in the specific pathways at initial colonization stage. (**B**) Venn diagram between the hub genes associated with YNM158 and DEGs in the specific pathways at infection stage. (**C**) DEGs Venn diagram between the initial colonization stage and infection stage in the same pathways. (**D**) Venn diagram between the hub genes associated with YNM158 and DEGs in both stages. (**E**–**J**) The relative expression of the core genes in YM158 and YNM158. *: *p* < 0.05; **: *p* < 0.01 by Student’s *t*-test.

Venn diagrams were drawn between the wheat DEGs at different infection stages and the hub genes positively associated with YNM158. At the initial colonization stage, 120 DEGs were obtained from specific enrichment pathways (Table S15), of these, 13 DEGs were associated with the FHB resistance of YNM158 (Figure 7A and Table S16). At the infection stage, 830 DEGs were obtained from a specific pathway (Table S17), of which 10 DEGs were related to the FHB resistance in YNM158 (Figure 7B and Table S16). In the same pathways in both stages mentioned above, 202 DEGs (Figure 7C and Table S18) were identified, 21 of which may be related to the FHB resistance of YNM158 (Figure 7D and Table S16).

To verify the correlation between the core genes and the stages of FHB resistance, the selected genes were verified using qPCR. Compared with the expression in YM158 at 0 hpi, the expression levels of six wheat genes in YNM158 were up-regulated after *F. graminearum* infection (Table 3). Of these, *TraesCS4D03G0528700* and *TraesCS4B03G0573000* belonged to the phosphatidylinositol-signaling system and protein processing in the endoplasmic reticulum pathway, respectively. The expression patterns of both genes were the same at the initial colonization stage and were substantially up-regulated in YNM158. *TraesCS4D03G0528700* showed the highest expression level at 2 hpi (Figure 7E), whereas *TraesCS4B03G0573000* reached its highest level at 8 hpi (Figure 7F). *TraesCS2D03G0030700* and *TraesCS7D03G0466200* encode the nucleotide-binding site-leucine-rich repeat (NBS-LRR) disease resistance protein and 3-ketoacyl–CoA synthase, respectively, both of which belong to the plant–pathogen interaction pathway. However, their expression patterns differed slightly. The expression of *TraesCS2D03G0030700* was substantially up-regulated after *F. graminearum* infection and the expression of this gene in YNM158 was always higher than that in YM158 (Figure 7G). Although *TraesCS7D03G0466200* was also induced after *F. graminearum* infection, the expression of *TraesCS7D03G0466200* in YNM158 was substantially higher than in YM158 until 48 hpi (Figure 7H). In addition, we found that the expression of one gene involved in the biosynthesis of secondary metabolites and the MAPK-signaling pathway was induced by *F. graminearum*. The qPCR results indicated that these two genes may mediate the resistance of YNM158 to FHB during the initial colonization and infection stages. *TraesCS7A03G1308100* encoded hydroxycinnamoyl–CoA shikimate, the expression of which, in YNM158, was substantially higher than that in YM158 from 8 h after *F. graminearum* infection (Figure 7I). In contrast, the expression of *TraesCS1A03G0718100* was substantially up-regulated at 0.5 hpi in YNM158. However, there was little difference in the expression of this gene between YM158 and YNM158 after 48 hpi (Figure 7J). These results suggest that the introduction of chromosome 7EL fragments may affect the disease-resistance pathway in wheat, thereby improving FHB resistance.

## 3. Discussion

### 3.1. YNM158 Can Be Effectively Applied in FHB Improvement in Wheat Breeding

Breeding and applying FHB-resistant varieties in wheat production is an effective way of controlling the destructive disease. However, long-term intraspecific cross-breeding of wheat has reduced the range of genetic variation among varieties and has resulted in poor resistance to FHB, whereas the related wild species and genera of wheat carry many FHB-resistant genes. For example, *Fhb3* on chromosome 7Lr#1S of *Leymus racemosus*, *Fhb6* on chromosome 1E(ts)#1S of *Elymus tsukushiensis*, and *Fhb7* on chromosome 7el_2_ of *Th. ponticum* were reported to have major resistance to FHB [6,7,8,9]. In addition, the 1Y^c^ and 3S^c^ chromosomes of *Roegneria ciliaris* [21], the 1E and 7E chromosomes of diploid *Th. elongatum* [12,13], chromosome 3St of *Elymus repens* [22], and 7M^g^ chromosome of *Aegilops geniculata* [23] also possess FHB-resistant genes. Although the introduction of alien chromosomes can improve resistance to FHB, it also introduces some genetic linkages, which makes the agronomic characteristics of most foreign germplasms poor and difficult to directly use in wheat breeding for FHB resistance. Therefore, to fully utilize wheat-related species in wheat breeding for FHB resistance, it is necessary to create small fragment translocation lines to develop new varieties with increased FHB resistance and no yield penalties. The *Fhb7* from *Th. ponticum* was introduced into cultivated wheat using small segment translocation lines and is used in wheat breeding for FHB resistance [9].

The diploid form of tall wheatgrass, *Th. elongatum*, has a high level of FHB resistance and has been used to increase FHB resistance in the wheat strain, Chinese Spring, by translocation development [19,24]. In this study, we successfully established translocation lines with small fragments of chromosome 7EL from diploid *Th. elongatum* using physical radiation, one of which has excellent agronomic traits and high resistance to wheat FHB (YNM158). Contrary to previous reports, the translocation in YNM158 occurred on chromosome 4BS, a non-compensative translocation, and the reason for this phenomenon was that chromosome translocation was induced by ionizing radiation; the breakage and reconnection of wheat and alien chromosomes were random, so most of them were uncompensated translocations. Interestingly, the survey results of agronomic traits for two consecutive years showed that YNM158 did not perform poorly because of a non-compensatory translocation line, such as the genetic instability of exogenous chromosomes, high plant height and poor fertility. The reasons for this are worthy of further investigation. Previous studies have shown that non-compensatory translocation lines have important utility in wheat breeding. For example, the small fragment translocation line 5VS–6AS·6AL can be used to improve the quality of wheat soft grains [25]; the 3A–7J^s^ translocation line can be used to improve wheat stem rust resistance [26]; and two homozygous translocation lines, T1AS·1AL–6VS and T4BS·4BL–6VS-4BL, carrying *Pm21*, can be used to enhance powdery mildew resistance in wheat [27]. Therefore, we propose that the translocation line YNM158, which contains a small fragment of chromosomes 7EL, has good application prospects in breeding wheat for resistance to FHB.

### 3.2. Transcriptome Analysis Validated That Glutathione Is One of the Important Contributors to FHB-Resistance Roles in the Pathogen Infection Stage

A decrease in the cost of the technique and widespread implementation have made transcriptome analysis a valuable tool for investigating the molecular mechanisms underlying cereal resistance to fungal infections. In the current study, glutathione metabolism was most enriched in YNM158 after *F. graminearum* infection during the infection stage (Figure 5D). Plants respond to fungal infections by activating defense genes, including the production of reactive oxygen species (ROS), which can enhance the strength of plant cell walls to resist pathogen invasion and colonization [28,29]. However, when large amounts of ROS accumulate in plants, they cause oxidative stress, damage plant cells, and lead to cell dysfunction and even death.

Currently, the enzymes involved in the antioxidant defense system can be divided into two groups: (i) enzymatic antioxidants, such as superoxide dismutase (SOD), catalase (CAT), ascorbate peroxidase (APX), guaiacol peroxidase GPX, glutathione reductase (GR), monodehydroascorbate reductase (MDHAR), and dehydroascorbate reductase (DHAR), and (ii) non-enzymatic antioxidants, such as ascorbic acid (AA), reduced glutathione (GSH), α-tocopherol, carotenoids, plastoquinone/ubiquinone, and flavonoids [30]. Early research has reported that the ascorbate–glutathione (AsA–GSH) cycle is an important method for removal of ROS in plants. Of these, APX is the key enzyme in this cycle and utilizes AsA as an electron donor to reduce H_2_O_2_ to water and prevent the accumulation of toxic levels of H_2_O_2_ in photosynthetic organisms under stress conditions [31]. Glutathione (GSH) participates in various metabolic processes and is an essential component of the antioxidative and detoxification systems in plant cells [32]. GSH can be used as both a reducing agent and strong nucleophile, participating in the elimination of reactive oxygen species (ROS) through thiol-disulfide redox reactions and in the detoxification of various heterogeneous organisms through conjugation reactions, respectively [33].

GSH is oxidized to glutathione (GSSG) during ROS clearance. To maintain the balance of GSH content in plants, the GR enzyme effectively and expeditiously reduces GSSG to GSH. It can be seen that the GR enzyme plays a very important role in clearing ROS and maintaining the content of GSH in plants. For example, the overexpression of *GR* gene from *Haynaldia villosa* in wheat can increase its resistance to powdery mildew [34]. In the current study, we found that the expression of some APX-encoding and GR-encoding genes in YNM158 was up-regulated during infection (Figure 5D). Therefore, we suggest that glutathione may play a key role in ROS-mediated resistance to FHB in wheat.

Glutathione S-transferase (GST) are a group of multifunctional enzymes widely present in plants and play important roles in plant secondary metabolism [35], growth and development [36], and biotic and abiotic stress responses [37]. One of its most important functions is the inactivation of toxic compounds. GST can form complexes with glutathione (GSH) by catalyzing hormones and toxins to inactivate or eliminate the toxicity of many substances and expel them into the body under the action of relevant transporters [38]. These results suggest that GST plays a crucial role in plant disease resistance. For example, *NbGSTU1* increases the resistance to *Colletotrichum destructivum* in *Nicotiana benthamiana* [39]. The lack of *GSTU13* function results in enhanced susceptibility toward several fungal pathogens in *Arabidopsis thaliana* [40]. Overexpression of *LrGST5* in tobacco can improve the resistance of transgenic plants to *F. oxysporum* [41]. *TaGSTU6* interactions enhance wheat resistance to powdery mildew but not wheat stripe rust [42].

Wheat infected with FHB can be contaminated with various mycotoxins, especially deoxynivalenol (DON) [43]. It has been reported that GSH can form GSH–DON conjugates under the catalysis of GST to reduce the accumulation of DON and protect plants from toxicity. For instance, *Fhb7* and *FhbRc1*, encoding glutathione S-transferase, enhance the resistance to FHB in wheat [9,44]. In this study, the expression of GST-encoding genes, including *TraesCS1A03G0109100*, *TraesCS3D03G0946300*, *TraesCS4D03G0493500*, *TraesCS5B03G0050700*, *TraesCS5A03G0730500*, *TraesCS5B03G0770700*, and *Tel7E01G1020600*, was substantially up-regulated after infection with *F. graminearum* in YNM158. Among them, the expression of the *Fhb7* homolog *Tel7E01G1020600* increased sharply at 72 hpi and was tenfold higher than that in non-infected cells (Figure 3E). It can be seen that GST is one of the important contributors to FHB resistance roles in the pathogen infection stage. However, the *Tel7E01G1020600* in YNM158 was derived from diploid *Th. elongatum*, which is not consistent with the origin of *Fhb7*. Therefore, whether *Tel7E01G1020600* in YNM158 has the same disease resistance function as *Fhb7* requires further investigation.

### 3.3. Other Genes from the 7EL Fragment in YNM158 Might Also Be Involved in Increasing FHB Resistance Especially in the Pathogen Initial Colonization Stage

The DON toxin is an important fungal pathogen produced when *F. graminearum* infects wheat. It can synthesize large amounts of *F. graminearum* along the inflorescence axis and promote disease expansion. However, some studies have reported that when the pathogen initially infects wheat anthers, there is no DON synthesis signal, and only when the disease spreads along the inflorescence axis from the inoculation point does DON begin to be synthesized in large quantities by the pathogen [45,46]. It can be seen that DON can help the pathogen spread along the wheat spike axis, but it is not necessary for its initial infection [47]. During the process of long-term co-evolution of plants and pathogens, a series of complex defense mechanisms have gradually formed. Pathogen-associated molecular pattern (PAMP)-triggered immunity (PTI) is the first line of defense against plant innate immunity and is mediated by pattern recognition receptors (PRRs). PRRs are divided into two types: receptor-like kinases (PLKs) and receptor-like proteins (PLPs). Many PLKs have been shown to play key roles in wheat disease resistance. For example, Sun et al. [48] reported that the repeat receptor-like kinase-encoding gene, *TaBIR1*, contributes to wheat resistance against *Puccinia striiformis f. sp. tritici* by mediating ROS production and callose deposition, and the cysteine-rich receptor-like kinase, TaCRK3, contributes to the defense against *Rhizoctonia cerealis* in wheat by directing anti-fungal activity and heightening the expression of defense-associated genes in the ethylene-signaling pathway [49].

RLKs have also been found to contribute to grain resistance to *Fusarium* resistance in cereals. For instance, Thapa et al. [50] identified two homologous genes on barley chromosome 6H (*HvLRRK-6H*) and wheat chromosome 6DL (*TaLRRK-6D*), respectively, which could enhance cereal resistance to FHB disease. Arabidopsis senses *Fusarium* elicitors during early immune responses to extracts from *Fusarium* spp. via a novel receptor complex encoded by the leucine-rich repeat receptor-like kinase MDIS1-interacting receptor-like kinase 2 (MIK2) at the cell surface [51]. Interestingly, we also identified several RLK-encoding genes on the 7EL fragment, and their expression was significantly up-regulated at the initial colonization stage after *F. graminearum* inoculation, such as *Tel7E01G943900*, which was substantially up-regulated at 0.5 hpi (Figure 3F).

During immune responses, plants have developed a number of disease-resistance mechanisms to resist nutrient uptake by pathogens that involve sugar transport, metabolism, and signal transduction. Previous studies have shown that hexose released by cell wall invertase (CWIN) not only acts as a signal molecule to trigger the expression of disease-resistance-related genes, but is also an essential metabolite and energy source for the synthesis of antioxidant compounds and defense molecules, such as salicylic acid and callose [52,53,54]. For example, *AtSTP4-* and *Atβfruct1*-encoding monosaccharide transporter and CWIN, respectively, are both induced in *Arabidopsis* during parasitic infection by fungus [55]. Chang et al. [56] reported that silencing the hexes transporter-encoding gene, *PsHXT1*, in wheat stripe rust can substantially inhibit the pathogenicity of bacteria. In the current study, the expression of the monosaccharide-sensing protein-encoding gene, *Tel7E01G980900*, was substantially up-regulated within 8 h of infection with *F. graminearum* and reached its highest level at 2 hpi in YNM158 (Figure 3G). The functions of monosaccharide-sensing proteins are similar to those of hexose transporters [57,58]. Therefore, we speculate that tests could also be conducted with CWINs to bring hexose back to host cells, reducing sugar availability to the pathogen, and thus improving host disease resistance. However, this must be confirmed in future studies.

It is well-known that *F. graminearum* is a kind of facultative trophic fungi. Therefore, wheat must use a series of defense mechanisms to resist pathogen infections at different stages. The introduction of 7EL chromosome fragments not only introduced the GST-encoding gene (which is one of the important contributors to DON detoxification) but also other genes that were upregulated at the initial colonization stage. These genes are also involved in increasing the FHB resistance. Therefore, an in-depth study of these genes may provide new insights into the molecular mechanisms underlying wheat resistance to FHB.

### 3.4. Introgression of the 7EL Fragment Altered the Gene Expression in Wheat after F. graminearum Inoculation

The introduction of alien chromosome fragments not only brings about resistance genes but also affects gene expression in normal chromosomes [59,60]. In the current study, the translocation of chromosomes affected the expression of wheat genes that were enriched in resistance pathways, including the phosphatidylinositol-signaling system, protein processing in endoplasmic reticulum, plant–pathogen interaction, and the MAPK-signaling pathway at different stages of *F. graminearium* infection.

When plants are infected with pathogens, phospholipase C (PLC) is rapidly activated by pathogen-associated molecular patterns (PAMPs) and effector proteins in plant cells [61]. Phosphatidylinositol 4-phosphate (PI4P) and phosphatidylinositol (4,5) bisphosphate [PI(4,5)P2] are catalyzed to produce inositol 2-phosphate (IP2), inositol 3-phosphate (IP3), and diacylglycerol (DAG). These are conserved compounds in pathogenic microbes that are perceived by immune receptors present in resistant plants [62,63]. Previous studies have reported that silencing and knock-out *SlPLC2* in tomatoes can reduce susceptibility to *Botrytis cinereal* [61,62]. *SlPLC6* plays a key role in both for Ve1-resistance-protein-mediated resistance to *Verticillium dahliae* and Pto/Prf-protein-mediated resistance to *Pseudomonas syringae* [64].

Salicylic acid (SA), jasmonate (JA), and methyl jasmonate can increase the expression of *OsPI-PLC* in rice (*Oryza sativa*) and improve the resistance of rice to *Magnaporthe oryzae* [65]. In the current study, the expression of some *PLC* genes in YNM158 was higher than in YN158 during the initial colonization stage, such as *TraesCS4A03G0225500* and *TraesCS4B03G0547100*. Moreover, the qPCR results showed that the expression of *TraesCS4D03G0528700*, which encodes phosphatidylinositol 4-phosphate-5 kinase (PIPK5), in YNM158, was higher than that in YM158 at the initial colonization stage (Figure 7B). We know that PIP5K is the catalytic enzyme for the synthesis of PI(4,5)P2. Shimada et al. [66] pointed out that the biosynthesis of PI(4,5)P2 is an important target for improving the defense ability of *Arabidopsis thaliana* against Colletotrichum, and its activity also determines the defense ability of *Arabidopsis thaliana* against Colletotrichum. Therefore, we speculated that PIP5K could affect the accumulation of PI(4,5)P2 in YNM1158 to participate in the PLC-mediated response to *F. graminearium* infection, thus affecting the colonization of *F. graminearium* to improve resistance to FHB during the initial stages of infection.

There are also defense-related proteins in plants that are synthesized by the rough endoplasmic reticulum (RER), so that when plants are attacked by the pathogen, the genes encoding endoplasmic reticulum (ER) chaperones are induced, such as the immunoglobulin-binding protein (BIP), heat-shock protein (Hsp), calreticulin (CRT), and protein disulfide isomerase (PDI)-encoding genes. Previous studies have shown that the Hsp are ER chaperones that play an indispensable role as molecular chaperones in the quality control of PRRs and intracellular resistance (R) proteins against potential invaders [67]. For example, Hsp90 is not only involved in the defense of many microbial pathogens by activating cytosolic R proteins containing a nucleotide-binding domain and a leucine-rich repeat but also participates in chitin responses and anti-fungal immunity in a chaperone complex with its co-chaperone, Hop/Sti1 [67,68].

In terms of specific diseases, cytoplasmic *Capsicum annuum* Hsp70 (CaHsp70) can enhance the resistance to *Xanthomonas campestris* pv. *vesicatoria* in pepper [69], GmHsp40 can increase soybean resistance to soybean *mosaic virus* [70], Hsp70 can enhance resistance to powdery mildew in cucumber under heat shock-induction [71], and the MeHsp90.9–MeSGT1–MeRAR1 chaperone complex interacts with MeATGs to trigger autophagy signaling to improve disease resistance to cassava bacterial blight [72]. In the present study, we found that the expression of *TraesCS4B03G0573000*, which encodes a heat shock protein in YNM158, was markedly upregulated after infection with *F. graminearium* at the initial colonization stage, which was opposite to the expression pattern observed in YM158 (Figure 7F). Typically, the expression of genes encoding ER chaperones precedes the expression of genes encoding pathogenesis-related (PR) proteins [73]. Therefore, we inferred that the expression of genes encoding the Hsp protein was rapidly induced after infection with *F. graminearium* in YNM158, thus activating the defense mechanism earlier, inducing programmed cell death, affecting the colonization of pathogens, and making plants resistant to disease. This provides a new idea for further research on the mechanism of FHB resistance.

Reactive oxygen species (ROS) are important signaling molecules in defense responses during plant–pathogen interactions and are mainly produced by respiratory burst oxidase homologs (RBOHs) [74]. In Arabidopsis, *AtRBOHD* and *AtRBOHF* are responsible for ROS production during pathogen attacks [75]. In *Nicotiana benthamiana*, *NbRBOHA* and *NbRBOHB* silencing led to reduced ROS production and reduced resistance to infection by the potato pathogen, *Phytophthora infestans* [76]. Phosphorylation is known to be one of the essential mechanisms of RBOHD activation and is also transcriptionally activated by some kinases, such as MAPK cascades, and the transcriptional regulation of RBOHs may play a key roles in subsequent ROS bursts after turnover of the plasma membrane-localized RBOHs used for the first burst [77]. For example, Yamamizo et al. [78] reported that MAPK is involved in inducing the response of potato *StRBOHC* and *StRBOHD* genes in response to pathogen signals in potato, and Asai et al. [79] illustrated that the MAPK cascade, MEK2–SIPK, regulates the oxidative burst resulting from the induction of *RBOHB* expression in resistance to *P. infestans* and *Colletotrichum orbiculare* in *N. benthamiana*. Here, we found that the expression of an RBOH-encoded gene, *TraesCS1A03G0718100*, was up-regulated after *F. graminearum* infection in YNM158 (Figure 7J), as well as some genes encoding MAPK (Figure 5G). Therefore, we hypothesized that the MAPK in YNM158 may be involved in inducing the RBOH gene response to resistance against *F. graminearum*. However, this requires further investigations.

## 4. Materials and Methods

### 4.1. Plant Materials

*Triticum aestivum* ‘Chinese Spring’ (CS), ‘Sumai3’ (SU3), ‘Annong8455’ (AN8455), and ‘Yangmai158’ (YM158) were maintained at Yangzhou University, China. YM158 was pollinated with the ^60^Co-γ-irradiated pollen of the long-arm translocation line TW-7EL1 (T7BS·7EL) of chromosome 7E with excellent FHB resistance developed in previous studies [80]. The chromosomes of the F_1_ generation plants were identified using GISH and plants containing 7EL chromosome structure variations were selected for backcrossing with YM158. After six generations of self-breeding, a small fragment of the 7EL chromosome translocation line, Yangnongmai158 (YNM158), was selected.

### 4.2. Cell Cycle Synchronization and Preparation of Mitotic Chromosomes

Cell cycle synchronization and slide preparation were performed as described by Lei et al. [81], with minor modifications. Seeds were soaked in water for 3–5 h and germinated on moist filter paper for 2 d in the dark at 25 °C. When the roots had grown to approximately 2.5 cm length, they were treated with 2 μmol/L amiprophosmethyl (APM) for 2.5 h. The root tips were then cut and placed in a nitrous oxide gas chamber for 1 h. The root tips were then fixed in ice-cold 90% acetic acid for 8 min, washed with sterile double distilled water (ddH_2_O), and stored in 70% ethanol at −20 °C until use. For slide preparation, the root tips were washed with ddH_2_O for 5 min. The apical meristems of the roots were cut and incubated in 25 μL of enzyme solution containing 2% cellulase Onozuka R-10 (Yakult Pharmaceutical, Tokyo, Japan) and 1% pectolyase Y23 (ICN) for 1 h at 37 °C in a water bath. Meristems were separated with a needle in 50 μL of 100% acetic acid and immediately dropped onto microscope slides using a pipette at a height of approximately 10 cm and then placed in a wet box for about 20 min. The number and location of chromosomes were observed and recorded under the phase contrast objective (Nikon 80i, Nikon, Tokyo, Japan), and the well-prepared slides were stored in a −70 °C refrigerator until use.

### 4.3. Genomic In Situ Hybridization (GISH) and Fluorescence In Situ Hybridization (FISH) Analysis

GISH and non-denaturing (ND)-FISH methods followed those described previously [82]. The total genomic DNA from *Th. elongatum* was labeled with digoxigenin-12-dUTP using the Nick Translation method and was used as a probe for GISH. Repetitive sequences of oligo-pSc119.2 and oligo-pAs1 were synthesized as probes. The 5′ ends of oligo-pSc119.2 and oligo-pAs1 were labeled using 6-carboxyfluorescein (6-FAM) and 6-carboxytetramethylrhodamine (Tamra), respectively. The labeled probes were dissolved in 2 × SSC and 1× TE buffer (pH 7.0) and dropped onto prepared slides. Next, the slides were covered with a coverslip, placed in a humidified hybridization cassette at 37 °C for 10 h, and then transferred to 2 × SSC for 2 min at room temperature. Finally, the slides were quickly dried and 6.5 μL DAPI was added to each slide (Vector, No. H-1200). After the ND-FISH analysis, the slides were washed in 2 × SSC for 2 min at room temperature. After drying the same slides were subjected to GISH. Hybridization signals were observed using a fluorescent microscope and images were obtained with a CCD camera (Color Cooled Digital DS-Fi1c, Nikon 80i, Nikon, Tokyo, Japan).

### 4.4. Evaluation of Disease Resistance

The translocation line YNM158 and its hybrid offspring were screened for FHB resistance in the field and greenhouse in 2021 and 2022, respectively. In this study, a single-floret inoculation method was used to assess FHB resistance. The monosporic isolate F0609 of *F. graminearum*, commonly found in the middle and lower reaches of the Yangtze River in China, was kindly provided by Professor Huaigu Chen, Jiangsu Academy of Agricultural Sciences, Nanjing, China. At the early flowering stage, the central spikelet was injected into 10 µL fungal suspension (50,000 spores/mL), and at least three spikes from each plant were injected. Following inoculation, the plants were incubated for 72 h for FHB development. After 21 d, all infected spikelets per inoculated spike were counted. Wheat cultivar, An8455, served as the susceptible control, and SU3 served as the resistant control in the field and greenhouse trials. One in both the field and greenhouse.

### 4.5. De Novo Assembly of RNA-Seq Reads and Quantifying Gene Expression

The transcriptome analysis was performed at eight time points after inoculation with *F. graminearum*: 0 hpi, 0.5 hpi, 2 hpi, 8 hpi, 24 hpi, 48 hpi, 72 hpi, and 96 hpi (three repetitions per time point). After inoculation, three spikes were randomly selected each time and mixed to extract RNA. Total RNA was extracted using a Trizol reagent kit (Invitrogen, Carlsbad, CA, USA) according to the manufacturer’s protocol. The RNA quality was assessed on an Agilent 2100 Bioanalyzer (Agilent Technologies, Palo Alto, CA, USA) and checked by using RNase-free agarose gel electrophoresis. The mRNA was enriched using oligo (dT) beads. Enriched mRNA was fragmented and used as the template for cDNA synthesis. The cDNA fragments were sequenced using an Illumina HiSeq2500 by Gene Denovo Biotechnology Co (Guangzhou, China). For analysis after sequencing, refer to the article published by Dai et al. [83]. The genomes of Chinese Spring (IWGSC RefSeq v2.1) and *Th. elongatum* (GWHABKY00000000) were used as reference genomes. The sequences obtained were submitted on the sequence read archive (BioProject ID: PRJNA1011388).

### 4.6. Quantitative Real-Time Polymerase Chain Reaction

The RNA used for transcriptome sequencing was provided by the company and used in subsequent reverse transcriptase experiments. The cDNA was generated from 1 μg of total RNA and synthesized using the HiScript 1st Strand cDNA Synthesis Kit (Vazyme, Nanjing, China). Each qPCR reaction mixture was 10 μL, containing 5 μL of 2 × SupRealQ Purple Universal SYBR qPCR Master Mix (Vazyme), 1 μL of a mix solution of target gene primers (10 mm), 1 μL of diluted first-strand cDNAs, and 3 μL of double-distilled H_2_O. The qPCR was performed under the following program: 94 °C for 5 min, and then 40 cycles: 94 °C for 10 s followed by 60 °C for 30 s. For the melt curve analysis, the following program was included after 40 cycles: 95 °C for 10 s followed by 60 °C for 30 s and a constant increase from 60 to 95 °C. The relative expression levels were determined using the −2^−ΔΔCt^ method. The qPCR assays were performed using three independent biological samples pretreatments and three technical replicates per sample. The primers used in this analysis are listed in Supplementary Table S19.

### 4.7. Data Analysis

All data were statistically analyzed using the IBM SPSS Statistics 25 software with pairwise comparisons of LSD to identify differences. Data conforming to normal distribution and homogeneity of variance were analyzed using one-way analysis of variance (ANOVA); otherwise, the Kruskal–Wallis one-way ANOVA was used. Different letters indicate significant differences (*p* < 0.05). GraphPad Prism 8 software was used to generate the figures.

## 5. Conclusions

FHB is a devastating wheat disease that seriously affects wheat yield and quality. Many laboratories worldwide have conducted studies on wheat resistance to FHB. It has been proven that the most economical and effective way to resist the damage caused by wheat FHB is to mine genes with high resistance to FHB and to breed new varieties that are resistant to FHB. In this study, the translocation line, YNM158, carrying the 7EL chromosome fragment obtained using distant hybridization not only had excellent resistance to FHB, but also had stable agronomic traits that could potentially be used in FHB resistance breeding. Transcriptome analysis indicated that the 7EL chromosome fragment not only carried genes that could detoxify DON but also contained genes that could affect the colonization of *F. graminearum* during the early stage of infection. In addition, introgression of the 7EL fragment altered gene expression and activated a specific resistance pathway in YNM158 after *F. graminearum* inoculation. YNM158 may have a variety of molecular mechanisms of action against *F. graminearum* infection and shows high resistance to the FHB phenotype. Therefore, these results not only provide a new germplasm for wheat resistance to FHB but also elucidate the molecular mechanism of wheat resistance and provide a new way for breeding new varieties with high resistance to FHB.

## Figures and Tables

**Table 1:** Mean percentage of diseased spikelets (PDS) in different environments.

Lines	2020–2021		2021–2022	
	Field	Greenhouse	Field	Greenhouse
**YNM158**	4.81% ± 0.19 d	5.18% ± 0.37 c	5.21% ± 0.23 d	5.27% ± 0.20 d
**SU3**	6.49% ± 1.79 d	7.38% ± 2.89 c	4.36% ± 0.22 d	5.06% ± 0.21 d
**AN8455**	83.42% ± 10.36 a	75.83% ± 14.69 a	87.88% ± 5.51 a	60.19% ± 6.24 a
**YM23**	28.79% ± 6.41 c	53.69% ± 5.15 b	15.54% ± 3.54 c	21.87% ± 7.05 c
**YM158**	47.01% ± 11.92 b	-	31.80% ± 12.91 b	45.09% ± 8.21 b

Note: The data were statistically analyzed by Kruskal–Wallis one-way ANOVA. Pairwise comparisons were completed using LSD. Different letters show significance at *p* < 0.05.

**Table 2:** The gene was verified by qPCR.

Gene ID	Gene Description
Tel7E01G1002700	Lysine ketoglutarate reductase trans-splicing-like protein (DUF707)
Tel7E01G1013700	Galactoside 2-alpha-L-fucosyltransferase
Tel7E01G1020600	Glutathione S-transferase
Tel7E01G211400	Protein kinase
Tel7E01G899900	NF-X1-type zinc finger protein NFXL1
Tel7E01G905000	Disease resistance protein (NBS-LRR class) family
Tel7E01G934300	Carbonic anhydrase
Tel7E01G939300	Receptor-like kinase
Tel7E01G941500	Carboxypeptidase
Tel7E01G943900	Receptor-like kinase
Tel7E01G946300	Blue copper binding protein
Tel7E01G980900	Monosaccharide-sensing protein 2

**Table 3:** The core genes verified by RT-PCR at different infection stages.

Gene ID	Pathway	Gene Description
Initial colonization stage		
TraesCS4D03G0528700	Phosphatidylinositol signaling system	Phosphatidylinositol-4-phosphate 5-kinase family protein
TraesCS4B03G0573000	Protein processing in endoplasmic reticulum	70 kDa heat shock protein
Infection stage		
TraesCS2D03G0030700	Plant–pathogen interaction	NBS-LRR disease resistance protein
TraesCS7D03G0466200	Plant–pathogen interaction	3-ketoacyl–CoA synthase
Both stage		
TraesCS7A03G1308100	Biosynthesis of secondary metabolites	Hydroxycinnamoyl–CoA
TraesCS1A03G0718100	MAPK signaling pathway–plant	Respiratory burst oxidase-like protein

## Data Availability

The genome sequences of Chinese Spring were downloaded from the website: https://wheat-urgi.versailles.inra.fr/Seq-Repository/Assemblies. The data was accessed on 24 April 2021. The genome sequences of *Th. elongatum* were downloaded from the website: https://ngdc.cncb.ac.cn/gwh/Assembly/965/show. The data was accessed on 10 March 2020. The transcriptome sequencing data generated in this study have been deposited in the NCBI Sequence Read Archive (SRA) database under accession number PRJNA1011388. And the datasets generated or analyzed during this study are included in this article and its additional file or are available from the corresponding author on reasonable request.

## Supplementary Materials

The following supporting information can be downloaded at: https://www.mdpi.com/article/10.3390/ijms25179452/s1.
