# Supplementary material for: Using Transcriptomics to Determine the Mechanism for the Resistance to Fusarium Head Blight of a Wheat-Th. elongatum Translocation Line
Source: Int J Mol Sci. 2024 Aug 30;25(17):9452. doi: 10.3390/ijms25179452 (PMC11395471; doi:10.3390/ijms25179452)
Supplement: Supplementary file 1 [file ijms-25-09452-s001.zip › Supplementary Figure.pdf]

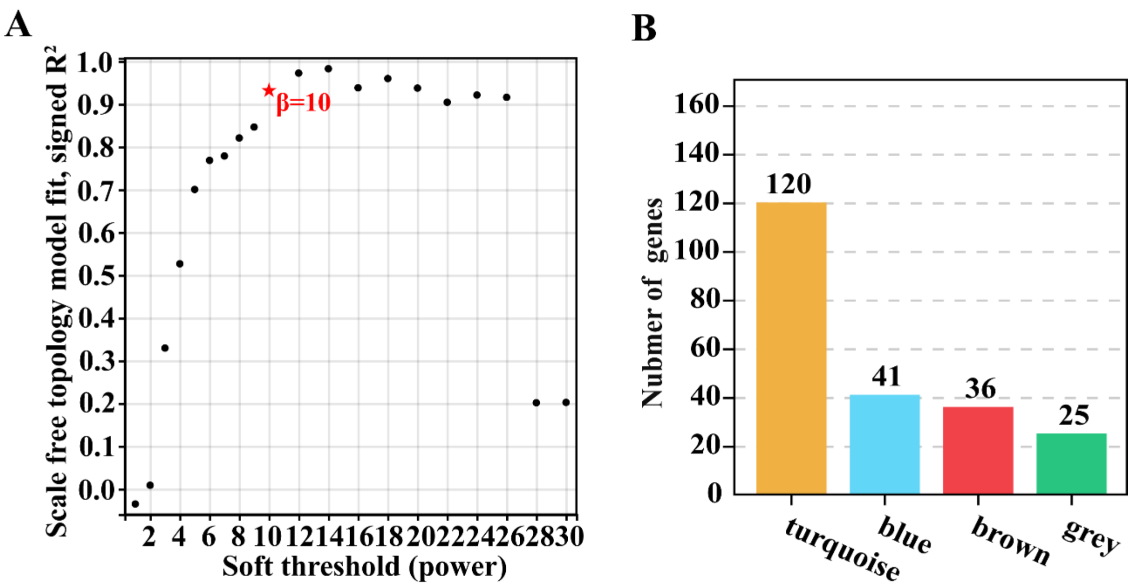

Fig. S1 **Determination of soft threshold.** (A) The x-axis represents the soft threshold β. (B) The number of genes clustered in the four modules.
